# How Does *Saccharomyces cerevisiae* DSM 34246 (Canobios-BL) *var. boulardii* Supplementation Impact the Fecal Parameters of Healthy Adult Dogs?

**DOI:** 10.3390/vetsci12010045

**Published:** 2025-01-10

**Authors:** Nicolò Lonigro, Francesca Perondi, Natascia Bruni, Mauro Bigliati, Annalisa Costale, Elena Pagani, Ilaria Lippi, Alice Melocchi, Lucia Zema, Giorgia Meineri, Elisa Martello

**Affiliations:** 1 Department of Drug Science and Technology, University of Turin, 10124 Turin, Italy; nicolo.lonigro@unito.it (N.L.); annalisa.costale@unito.it (A.C.); 2 Department of Veterinary Science, University of Pisa, 56122 Pisa, Italy; f.perondi87@gmail.com (F.P.); ilaria.lippi@unipi.it (I.L.); 3 Candioli Pharma S.r.l., 10092 Turin, Italy; natascia.bruni@candioli.it (N.B.); bigliativet@virgilio.it (M.B.); 4 Monge & C. S.p.A, 12030 Cuneo, Italy; elena.pagani@monge.it; 5 PhormulaMI Lab, Department of Pharmaceutical Sciences, University of Milan, 20133 Milan, Italy; alice.melocchi@unimi.it (A.M.); lucia.zema@unimi.it (L.Z.); 6 Department of Veterinary Sciences, School of Agriculture and Veterinary Medicine, University of Turin, 10095 Turin, Italy; giorgia.meineri@unito.it; 7 Division of Epidemiology and Public Health, School of Medicine, University of Nottingham, Nottingham NG5 1PB, UK

**Keywords:** pet, probiotic, feed supplements, yeast products, immunomodulation, gut health

## Abstract

Background: A supplement containing *Saccharomyces cerevisiae* DSM 34246 (Canobios-BL) *var. boulardii* was tested to demonstrate its efficacy as a gut health stabilizer in two breeds of dogs, West Highland White Terrier (WT) and German Shepherd (GS). Methods: A randomized double-blind controlled trial was performed on a total of 53 healthy adult dogs, 28 WT and 25 GS. The animals were randomly assigned to a control group (CTR: WT 14/28, GS 12/25) and to a treated group (SACC; WT 14/28, GS 13/25). The CTR group was fed with dry commercial food, while the SACC group was fed with the same food but supplemented with *S. boulardii* at the concentration of 5 × 10^9^ CFU/kg. The study lasted 35 days and included six evaluation time points (T_0_-T_5_). Body Weight (BW), Body Condition Score (BCS), Fecal Score (FS), FS measured with a penetrometer (FSp), Fecal Dry matter (DM), Fecal Humidity (UM), and Fecal IgA (IgA) were measured at each time point. Results: A significant improvement (*p* < 0.1) in BCS, FS, FSp, DM, UM, and IgA in the SACC group compared to the CTR for both WT and GS was reported. Conclusions: We found a positive effect of *S. boulardii* supplementation on the improvement of the fecal parameters and on the maintenance of good physiological as well as biological conditions in healthy adult dogs in breeding conditions.

## Simple Summary

This study tested *Saccharomyces cerevisiae* DSM 34246 (Canobios-BL) *var. boulardii* on adult dogs (West Highland White Terrier (WT) and German Shepherd (GS)) to evaluate its impact on gut health. A total of 53 healthy adult dogs were randomly assigned to control (CTR: WT 14/28, GS 12/25) and treated (SACC: WT 14/28, GS 13/25) groups. Both were fed a dry diet twice daily, with the additive given to the SACC group (5 × 10⁹ CFU/kg) and a placebo to the CTR. Over 35 days, body weight, body condition score, fecal parameters, and water intake were measured. Statistical analysis showed significant improvements in body condition, fecal parameters, and IgA, indicating *S. boulardii* positively affected gut health in dogs.

## 1. Introduction

The gastrointestinal (GI) tract is populated by a variety of microbes, which were recently demonstrated to play a major role in both human and animal health [1]. In fact, “the healthy gut” is overall linked to the welfare of the host not only because it is a vital organ for digestion and absorption of nutrients, but also as it is involved in the defense against pathogens and in the maintenance of the immune system [2,3].

The gut microbiota is essential for maintaining the homeostasis of the host, especially when there is a microbial imbalance in the GI tract, which might possibly lead to a change in the microbiome, influencing gut health [1,3]. The use of supplements for maintaining gut health is also increasing in veterinary medicine, as their administration has shown them to be effective and associated with limited side effects [4]. For example, probiotics—intended to be administered as live organisms able to provide health benefits to the host [3,4]—are largely used to maintain GI health and mitigate stress-dependent GI problems in animals [5]. Specifically, they support the barrier function of intestinal tissues and their regeneration [3,6].

Several bacteria have recently been identified as probiotics [7] and, for example, beneficial effects related to the supplementation of *Saccharomyces cerevisiae var. boulardii* in both animals [3,8,9,10] and humans [11,12] have been preliminary reported. Indeed, the administration of *S. boulardii* in humans affected by GI diseases, such as chronic diarrhea and inflammatory bowel disease (IBD), has demonstrated clinical effectiveness, indicating that such a probiotic might positively interfere with inflammatory conditions [4,11]. Thus, *S. boulardii’s* probiotic activity in humans has been linked with multiple effects, such as improvements in gut barrier function, pathogen competitive exclusion, the production of antimicrobial peptides, immune modulation, and trophic effects on the gut [4,13]. Similarly, *S. boulardii* recently started to be used as a probiotic in pets [2,3,14]. One study showed an improvement in the intestinal status and general health conditions of dogs suffering from chronic enteropathies when treated for 60 days with *S. boulardii* [14]. In addition, a different study involving healthy dogs in breeding conditions reported promising outcomes regarding intestinal microbiota and nutritional as well as stress status, resulting from the supplementation of *S. boulardii* for 42 days [3].

Based on the above-mentioned considerations, the aim of this randomized double-blind controlled trial was to demonstrate the efficacy of the feed additive *S. cerevisiae* DSM 34246 (Canobios-BL) *var. boulardii* as a “gut flora stabilizer” in healthy dogs.

## 2. Materials and Methods

### 2.1. Animals and Study Design

This randomized double-blind controlled trial included a group of 53 adult dogs (aged from 1 to 5 yr). In detail, 28 dogs were West Highland White Terriers (WTs) (10 males and 18 females), and the other 25 were German Shepherds (GS) (13 males and 12 females), selected from two ENCI-registered breeders in Italy.

Animals were randomly assigned to control (CTR: WT 14/28, GS 12/25) and treated (SACC: WT 14/28, GS 13/25) groups.

Both groups were fed with a commercial dry food diet for adult dogs (Monge Natural Superpremium Grain Free all breeds Adult Anchovies with Potatoes and Peas) two times a day, from at least 7 days before the beginning of the study.

The amount of daily food was calculated based on the following equation: (NRC, 2006): ME (kcal/day) = 110 × (kg BW)^0.75^.

A placebo (Maltodextrin powder) or a supplement containing *S. cerevisiae* DSM 34246 (Canobios-BL) *var. boulardii* (5.0 × 10^9^ CFU/kg) [15] was added to the pet food given to the CTR or to the SACC group, respectively, once a day for the whole duration of the study (35 consecutive days). The study was divided into five experimental times (from T_0_ to T_5_), with each time point separated from the previous and/or the following one by a seven-day interval. The number of animals and the length of the trial were set according to EFSA (European Food Safety Authority) regulations for registering a feed additive [15].

At the beginning of the study, a veterinarian checked the health status of the animals through a general physical examination. All the recruited animals were healthy with no underlying conditions or drugs administered in the past 15 days. In the case of a change in health status, pregnancy, pharmacological treatments, diet modification, pathological symptoms, and/or death, the dogs would have to be excluded from the study. Dogs were housed in kennels with two or three animals per cage. The cage area was 6 (±2) square meters in size, with an open space of the same size, considering the principles of animal welfare and the Italian regulations, thus avoiding any stress. The breeders were informed of the design of the study and signed a written informed consent form. The experimental procedures used were approved by the Bioethics Committee of the University of Turin, Italy (approval 156895, 14 April 2020).

### 2.2. Data Collection

All the tests aimed at evaluating the nutritional status of the dogs were performed by the same veterinarian, following standard guidelines [16]. Daily water intake (WI) in liters was reported at each time point by the breeders. Body Weight (BW), Body Condition Score (BCS), Fecal Score (FS), FS measured with a penetrometer (FSp), Fecal Dry matter (DM), Fecal Humidity (UM), and Fecal IgA (IgA) were selected as the parameters for this study. The BW (kg) was recorded at T_0_ (day 0), T_1_ (day 7), T_2_ (day 14), and T_3_ (day 21), T_4_ (day 28), and T_5_ (day 35). The BCS [17] was recorded in scores ranging between 1 and 9, with the points being assigned after a visual examination and palpation of the animal at T_0_ and T_5_. A score of 4 or 5 represents the ideal [17] (WSAVA 2013). Four fecal parameters, FS (1-7), FSp (1-7), DM (%), UM (%), and IgA (mg/g), were evaluated at each time point.

FS was determined by direct examination of fresh feces using a 7-point scale, with an ideal score between 3 and 4 [18,19].

The measurement of fecal hardness in kg/cm^2^, was performed on fresh stool with a Penetrometer 53220 FTA (GUSS Manufacturing, PTY Ltd., Cape Town, South Africa) using a technique already described by Davies and colleagues (Davies et al., 1986) [18]. The amount of stool used for each analysis was at least 40–50 g depending on the type and shape of the stool itself. The fecal hardness was defined as the mean of three measurements. Fecal hardness was converted using a validated scale to obtain the FSp.

For the assessment of the UM, a 5–10 g stool sample was weighed, dried in an oven at 105–110 °C for 20–24 h, and cooled down in a desiccator for 20–24 h. Then, the dried sample was weighed again. The final UM result was defined as the mean of two different measurements and expressed as percentage.

DM was calculated from the UM through the following method: DM = 100 − UM. The result was expressed as a percentage.

Lastly, the IgA level was measured on wet fecal samples using a specific ELISA kit (Dog IgA ELISA Quantitation Set, Bethyl Laboratories Inc., Montgomery, TX, USA).

### 2.3. Statistical Analysis

The statistical analysis conducted in the study varied depending on the type of parameter considered (categorical or numerical). In more detail, BW, FSp, IgA, DM, and UM were assessed using the analysis of variance (ANOVA) based on a repeated measures model, implemented through the mixed procedure (PROC MIXED MODEL SAS 9.4, 2013).

The statistical model was structured as follows:

y = μ + Si + Gj + Tk + GTjk + ejkn(1)

where y = dependent variable; μ = overall mean; Si = fixed effect of the sex (i = F; M); G = fixed effect of the treatment (j = 0.1); Tk = fixed effect of the kth time (k = 1.6). GTjk = fixed effect of the interaction between the jth treatment and kth time; ejkn = error.

Time was treated as a repeated measurement and replicated with groups as repeated subjects. The autoregressive covariance method was used for the covariance structure. Least square means were separated using Student’s *t*-test.

The BCSs and FSs of the CTR and SACC groups were compared using the Kruskal–Wallis test (UCLA) for both the overall experimental period and the supplementation period, using PROC NPAR1WAY (SAS 9.4). If significant results were found, multiple comparison analyses based on pairwise two-sample Wilcoxon comparisons were conducted. Test results from the two-tailed tests with *p*-values < 0.10 were considered significant.

## 3. Results

All the dogs involved in the trial were healthy during the study and did not need any drugs or supplements at any time. Moreover, they did not receive any pharmacological treatment in the 15-day period before the start of the study, and no change in feed administration or in relevant consumption was highlighted.

In Table 1, the effects of the supplementation of *S. boulardii* on BW and BCS by breed are summarized. In more detail, at the beginning of the evaluation (T_0_) the least square means (±SE) for the BW in the WT group were 8.2 ± 0.3 (CTR) and 7.50 ± 0.3 (SACC), while in the GS group they were 32.1 ± 1.6 (CTR) and 31.6 ±0.9 (SACC). The differences in BW between the SACC and CTR groups at T_0_ were not significant over time in both dog breeds and treatment groups.

Focusing on the BCS, the mean values were 5.6 ± 0.1 (CTR) and 5.5 ± 0.1 (SACC) in the WT group, while they were 5.5 ± 0.2 (CTR) and 5.6 ± 0.2 (SACC) in the GS group at the beginning of the study with no significant differences reported (Table 1). Interestingly, for both dog breeds, the BCSs of SACC group reduced significantly over time (i.e., from T_0_ to T_5_), but such a decrease was not evident in the CTR group (Table 1). Overall, significant differences were observed in WT and GS between CTR and SACC from T_1_ to T_5_ (WT) and T_2_ to T_5_ (GS).

The FSp in both breeds in the SACC group showed a statistically significant decrease over time, with a mean value near to 3 at the end of the study. The CTR group did not show a significant reduction in the values measured. The FSp was significantly different between the two groups (T_1_–T_5_) in both breeds, and in the SACC groups it significantly decreased from T_1_ to T_5_ (Table 2).

In the same way, the FS observed by the veterinarian showed a statistically significant decrease in the SACC groups with the progression of the trial, with a value near to 3 for most of the animals at the end of the study period (T_5_). In the WT group, the FS was different between the CTR and SACC groups at T_2_, T_4_, and T_5_ (Table 2), with the SACC group showing a significant decrease.

The DM measured for both breeds in the SACC group showed a statistically significant increase overtime from T_1_ (GS) or T_2_ (WT) to T_5_ and compared to the CTR group from T_1_ (GS) or T_2_ (WT) (Table 2).

The UM significantly decreased from T_1_ (GS) or T_2_ (WT) to T_5_ in the SACC group in both breeds (Table 2).

The Fecal IgA level in both breeds showed a significant increase from T_1_ to T_5_ in the SACC groups, while all the CTR groups did not show any significant increase. The IgA measurements pointed out statistically significant differences between groups at T_3_, T_4_, and T_5_ (Table 2).

Finally, the WI in the CTR and SACC groups within the same breed did not show any statistically significant variation during the whole study period (Table 3).

## 4. Discussion

The most novel research on pets emphasizes the benefits of yeast products on the modulation of the intestinal microbiota (i.e., with potential increases in *Bifidobacterium* or *Lactobacillus*), with these compounds being able to enhance immune function, reduce potentially pathogenic microorganisms, and improve the animal’s antioxidant status [7]. For example, *S. boulardii*, i.e., the probiotic yeast used in this study, can inhibit the colonization of pathogenic microorganisms, improve intestinal barrier function, and regulate immunity [13]. Very recently, its efficacy in promoting intestinal health and microbiome composition has also been demonstrated in kittens [2].

In the present study, *S. boulardii* was tested as a probiotic feed additive in healthy dogs for a period of 35 days. It did not cause any short-term adverse effects, as already reported by other authors [3,14]. At the beginning of the trial, all the dogs involved were healthy and no significant differences in the parameters selected as the outputs of the evaluation were highlighted.

In recent years, an increased use of probiotics in animal diets as supplements to maintain optimal gastro-intestinal health in both healthy pets and pets with disorders has been reported [1].

Moreover, yeast and yeast-based products have been shown to potentially promote gut health in both humans and animals [7]. However, there is still limited scientific literature discussing the impact of yeast products in dogs and cats, although the number of publications in this respect is slowly increasing [7].

*Saccharomyces cerevisiae* is one of the predominant yeast products used in various applications [7]. Various research works have demonstrated its beneficial effects on the intestinal health and microbiota of dogs, in both healthy animals [3] and in those with various gastrointestinal disorders [14]. Its administration improved dogs’ fecal consistency (higher FS) resulting in a higher DM [20] when compared to the control groups, although the values remained within the ideal score range in some of the studies [7,14,21].

In this work, at the end of the treatment period, no significant differences in BW were reported in the SACC groups compared to the CTR groups nor over time, while the BCS showed a significant variation in the treated versus untreated dogs. At T_0_ the two groups were similar in values (5.5–6), and then the BCS gradually decreased only in the treated groups, reaching a medium value between 4.5 and 5 at T_5_ [17]. This finding highlights the positive effect of the supplement on BCS as it remains within the physiological range reflecting a good maintenance of the nutritional condition in the two dog breeds, which are known to have the tendency to become overweight, as also previously reported [7].

In addition, the data collected demonstrated the positive impact of *S. boulardii* on all fecal parameters under evaluation (FS, Fsp, DM, UM, IgA) for both dog breeds.

Indeed, FS as well as FSp presented significant differences in the two groups; specifically, they decreased in the SACC one, which was characterized by harder feces compared to the CTR groups, whose values were between 3 and 4 (Table 2).

A significant improvement in the other two fecal parameters (DM and UM) was reported, further strengthening the positive effect of *S. boulardii* supplementation on fecal consistency due to the minor water content in the samples.

In this trial, water intake (WI) should not have modified the fecal consistency parameters, as all animals, regardless of their treatment group, had similar values throughout the trial. Moreover, there was no statistically significant variation in WI between the CTR and SACC groups in both breeds during the study. This result supports the positive effect of *S. boulardii* on the previously discussed fecal parameters.

Interestingly, the administration of *S. boulardii* led to a significant increase in fecal IgA in the SACC group (Table 2). Fecal IgA is a biomarker for intestinal immunity or inflammation in dogs and puppies [22] and has been recently proposed as a non-invasive marker of canine intestinal health [3,7,22]. Indeed, secretory IgA is the most important humoral protective immune factor in the intestine. It inhibits the adhesion, colonization, and penetration of microorganisms, as well as the absorption of food antigens [23]. The data collected in this work, consistent with findings from other studies, confirmed that the use of supplementation with yeast products in dogs increases ileal and Fecal IgA, indicating enhanced mucosal immunity and immunomodulatory properties [7,20,24]. This study has some limitations; specifically, it would benefit from a longer trial period, the inclusion of different dog breeds to avoid potential breed biases, and the evaluation of other blood and fecal inflammatory parameters. Moreover, further analysis of changes in the gut microbiota would be useful to further support the role of *S. boulardii* as a gut flora stabilizer in healthy dogs.

## 5. Conclusions

The supplementation of *S. boulardii* for 35 days, at the recommended dietary dosage of 5.0 × 10^9^ CFU/kg of feed in healthy dogs of different breeds (WH and GS), significantly improved the BCS, FS, FSp, DM, UM, and Fecal IgA parameters. Based on the results obtained, feed supplementation with the above-mentioned yeast product could also be recommended in clinical settings for an improvement in the fecal parameters of healthy dogs, also promoting an enhanced mucosal immunity. Further studies are recommended in dogs and cats suffering from gastrointestinal diseases.

## Figures and Tables

**Table 1:** Body weight (BW) and Body Condition Score (BCS) parameters measured in treated (SACC) versus untreated (CTR) West Highland White Terriers (WTs) and German Shepherds (GSs) at different times during the study.

		West Highland White Terrier (WT n = 28)			German Shepherd (GS n = 25)		
		CTR	SACC	*p*-Value	CTR	SACC	*p*-Value
BW (±SE) (kg)	T_0_	8.2 ± 0.3	7.5 ± 0.3	0.1	32.1 ± 1.6	31.6 ± 0.9	0.6
	T_1_	8.0 ± 0.3	7.6 ± 0.3	0.2	31.8 ± 1.6	32.0 ± 0.9	1.0
	T_2_	8.0 ± 0.3	7.4 ± 0.3	0.2	31.7 ± 1.6	31.8 ± 1.0	0.9
	T_3_	8.1 ± 0.3	7.5 ± 0.3	0.2	31.8 ± 1.6	31.9 ± 0.9	0.9
	T_4_	8.3 ± 0.3	7.6 ± 0.3	0.1	31.7 ± 1.5	32.1 ± 0.9	0.9
	T_5_	8.3 ± 0.3	7.7 ± 0.3	0.1	31.6 ± 1.5	32.1 ± 0.9	0.8
BCS (±SE) (1–9)	T_0_	5.6 ± 0.1	5.5 ± 0.1	0.7	5.5 ± 0.2	5.6 ± 0.1	0.5
	T_1_	5.5 ± 0.1	5.0 ± 0.1	<0.1	5.4 ± 0.1	5.2 ± 0.1	0.5
	T_2_	5.5 ± 0.1	4.9 ± 0.1	<0.1	5.4 ± 0.1	4.6 ± 0.1	<0.1
	T_3_	5.6 ± 0.1	4.9 ± 0.1	<0.1	5.3 ± 0.1	4.4 ± 0.1	<0.1
	T_4_	5.7 ± 0.1	4.8 ± 0.1	<0.1	5.3 ± 0.2	4.4 ± 0.1	<0.1
	T_5_	5.6 ± 0.1	4.6 ± 0.1	<0.1	5.2 ± 0.2	4.4 ± 0.1	<0.1

**Table 2:** Fecal Score (FS), FS measured with a penetrometer (FSp), Fecal Dry matter (DM), Fecal Humidity (UM), and Fecal IgA (IgA) parameters measured in treated versus untreated West Highland White Terriers (WTs) and German Shepherds (GSs) at different times during the study.

		West Highland White Terrier (WT n = 28)			German Shepherd (GS n = 25)		
		CTR	SACC	*p*-Value	CTR	SACC	*p*-Value
FS (±SE) (1–7)	T_0_	4.3 ± 0.2	4.3 ± 0.2	0.9	4.3 ± 0.4	4.2 ± 0.3	0.9
	T_1_	4.3 ± 0.2	4.1 ± 0.2	0.4	4.4 ± 0.2	3.8 ± 0.2	<0.1
	T_2_	4.3 ± 0.2	3.6 ± 0.2	<0.1	4.0 ± 0.2	3.4 ± 0.1	<0.1
	T_3_	4.1 ± 0.3	3.8 ± 0.2	0.4	4.6 ± 0.4	3.3 ± 0.2	<0.1
	T_4_	4.3 ± 0.3	3.6 ± 0.2	<0.1	4.8 ± 0.3	3.5 ± 0.1	<0.1
	T_5_	4.1 ± 0.2	3.4 ± 0.1	<0.1	4.3 ± 0.2	3.2 ± 0.1	<0.1
FSp (±SE) (1–7)	T_0_	4.5 ± 0.2	4.3 ± 0.3	0.7	4.4 ± 0.3	4.3 ± 0.3	0.7
	T_1_	4.5 ± 0.1	3.9 ± 0.2	<0.1	4.6 ± 0.2	3.9 ± 0.2	<0.1
	T_2_	4.6 ± 0.3	3.2 ± 0.2	<0.1	4.3 ± 0.2	3.3 ± 0.1	<0.1
	T_3_	4.6 ± 0.3	3.5 ± 0.2	<0.1	4.6 ± 0.2	3.3 ± 0.1	<0.1
	T_4_	4.6 ± 0.3	3.2 ± 0.1	<0.1	4.8 ± 0.3	3.3 ± 0.1	<0.1
	T_5_	4.3 ± 0.3	3.0 ± 0.2	<0.1	4.3 ± 0.2	3.1 ± 0.1	<0.1
DM (±SE) (%)	T_0_	30.4 ± 2.2	32.6 ± 2.8	0.5	29.2 ± 4.3	31.7 ± 2.8	0.6
	T_1_	32.3 ± 2.0	34.2 ± 1.5	0.6	32.5 ± 1.9	37.3 ± 1.3	<0.1
	T_2_	31.9 ± 2.1	42.6 ± 1.5	<0.1	36.1 ± 1.1	41.8 ± 1.4	<0.1
	T_3_	33.3 ± 2.9	39.8 ± 2.4	<0.1	36.1 ± 3.1	41.4 ± 2.1	<0.1
	T_4_	31.4 ± 2.7	42.5 ± 1.8	<0.1	34.6 ± 3.0	39.8 ± 1.7	<0.1
	T_5_	33.1 ± 2.0	44.5 ± 1.2	<0.1	37.9 ± 1.7	44.0 ± 1.2	<0.1
UM (±SE) (%)	T_0_	68.6 ± 1.1	65.9 ± 2.8	0.4	66.0 ± 4.3	67.1 ± 2.8	0.7
	T_1_	66.8 ± 1.0	64.5 ± 1.5	0.5	68.1 ± 2.0	61.5 ± 1.2	<0.1
	T_2_	67.1 ± 1.0	56.8 ± 1.5	<0.1	67.8 ± 1.2	57.5 ± 1.4	<0.1
	T_3_	65.8 ± 1.4	59.6 ± 2.4	<0.1	70.1 ± 3.1	57.9 ± 2.1	<0.1
	T_4_	67.8 ± 1.4	57.0 ± 1.8	<0.1	72.6 ± 2.9	59.3 ± 1.7	<0.1
	T_5_	65.9 ± 1.0	55.0 ± 1.2	<0.1	66.5 ± 1.7	55.7 ± 1.2	<0.1
IgA (±SE) (mg/g)	T_0_	0.6 ± 0.1	0.69 ± 0.1	0.3	0.57 ± 0.08	0.58 ± 0.04	0.8
	T_1_	0.6 ± 0.1	0.68 ± 0.1	0.2	0.51 ± 0.07	0.62 ± 0.04	0.3
	T_2_	0.6 ± 0.1	0.74 ± 0.1	0.2	0.51 ± 0.08	0.71 ± 0.06	<0.1
	T_3_	0.6 ± 0.1	0.8 ± 0.1	<0.1	0.50 ± 0.08	0.78 ± 0.07	<0.1
	T_4_	0.6 ± 0.1	0.9 ± 0.1	<0.1	0.52 ± 0.08	0.85 ± 0.07	<0.1
	T_5_	0.5 ± 0.1	1.0 ± 0.1	<0.1	0.50 ± 0.07	0.92 ± 0.08	<0.1

**Table 3:** Water intake (WI) expressed in liter (lt) measured in treated (SACC) versus untreated (CTR) dogs West Highland White Terriers (WTs) and German Shepherds (GSs) at different time points during the study.

		West Highland White Terrier (WT n = 28)		German Shepherd (GS n = 25)	
		CTR	SACC	CTR	SACC
WI (±SE) (Lt)	T_0_	0.38 ± 0.02	0.37 ± 0.02	1.58 ± 0.06	1.58 ± 0.05
	T_1_	0.38 ± 0.02	0.38 ± 0.02	1.60 ± 0.05	1.59 ± 0.08
	T_2_	0.39 ± 0.01	0.38 ± 0.02	1.57 ± 0.06	1.59 ± 0.08
	T_3_	0.37 ± 0.01	0.38 ± 0.02	1.57 ± 0.05	1.57 ± 0.04
	T_4_	0.38 ± 0.02	0.39 ± 0.01	1.60 ± 0.06	1.57 ± 0.04
	T_5_	0.38 ± 0.02	0.38 ± 0.02	1.58 ± 0.05	1.59 ± 0.05

## Data Availability

The data that support the findings of this study are available on request from the corresponding author (EM).
